# Treating rhinitis in the older population: special considerations

**DOI:** 10.1186/1710-1492-5-9

**Published:** 2009-12-01

**Authors:** Raymond G Slavin

**Affiliations:** 1 Department of Internal Medicine, Division of Immunobiology, Section of Allergy & Clinical Immunology, Saint Louis University School of Medicine, 1402 South Grand Boulevard, M157, Saint Louis, MO 63104, USA

## Abstract

Rhinitis in the elderly is a common but often neglected condition. Structural changes in the nose associated with aging, predisposes the elderly to rhinitis. There are a number of specific factors that affect medical treatment of the elderly including polypharmacy, cognitive dysfunction, changes in body composition, impairment of liver and renal function and the cost of medications in the face of limited resources. Rhinitis in the elderly can be placed in several categories and treatment should be appropriate for each condition. The most important aim is to moisten the nasal mucosa since the nose of the elderly is so dry. Great caution should be used in treatment with first generation antihistamines and decongestants. Medications generally well tolerated by the elderly are second generation antihistamines, intra-nasal anti-inflammatory agents, leukotriene modifiers and iprapropium nasal spray.

## 

Rhinitis is a common and bothersome condition in the elderly. Despite its importance, little attention is paid in the general medical literature. In the most recently published highly regarded geriatric text, rhinitis is not included in the index whereas rhinophyma is [1].

The number of Americans older than 65 years of age will increase from 35 million to 86 million by the year 2050 [2]. While the exact number of elderly patient with rhinitis is not known, it is believed that 40% of the general population experiences nasal symptoms [3]. It would be safe to say that the many changes that occur in the connective tissue and vasculature of the nose predisposes aging individuals to chronic rhinitis making the percentage of the elderly with nasal symptoms significantly higher than the general population [4].

The elderly have generalized decrease in body water content and, along with a degeneration of mucous-secreting glands; the effectiveness of the mucociliary system is reduced, resulting in symptoms of nasal stuffiness. In addition, a decrease in nasal blood flow leads to atrophy and drying of the nasal mucous membrane and increased mucous viscosity. Structural changes in the nose with age include atrophy of the collagen fibers and loss of elastic fibers in the dermis. Weakening of the upper and lower nasal cartilage, retraction of the nasal columella, and downward rotation of the nasal tip contribute to an increase in nasal airway resistance [3].

This article will deal with the special considerations of treating rhinitis in the older population. Appendix 1 lists the specific factors that may affect general medical treatment in the elderly.

The elderly patient is frequently being treated for a variety of medical conditions with a number of medications. The more medications that are prescribed the less likely the patient is to comply.

Aside from complying with directions for a large number of medications, the elderly patient frequently has cognitive dysfunction with a resultant decrease in memory.

A number of changes in body composition associated with growing older may effect distribution of particular medications. These changes include decrease in muscle mass, fat and body water.

Medications metabolized through the liver and kidney may be affected by decrease in function of the organ systems.

Finally, many elderly patients have limited financial resources and may simply not be able to afford the cost of the prescribed medications.

## Types of rhinitis in the elderly

Appendix 2 shows the various categories of rhinitis in the elderly. I will discuss each condition and the appropriate therapeutic approach.

Before going into specific treatment options, I would like to emphasize the main goal of treatment in most older patients; namely moistening the nasal mucosa. The nose of the elderly is dry for a number of reasons; a general decrease in the body water content; degeneration of mucous secreting glands, and a decrease in nasal blood flow. Nasal lavage with isotonic sodium chloride is usually the preferred method to reduce the nasal dryness and facilitate the clearing of thick mucous and crusts. Several randomized controlled studies on saline nasal irrigation suggest that it is a safe, effective, well-tolerated method to be used in inflammatory diseases of the nose [5].

## Allergic rhinitis

The incidence of allergic rhinitis decreases with age. The peak incidence of 37 per 1000 occurs at 10 to 15 years of age, with the incidence over 65 years of age being less than 3 per 1000 [6]. A significant decline in serum IgE levels occurs with aging in atopic individuals with a tendency for RAST and immediate-type skin test responses for selected antigens and histamine to decrease [7]. While allergic rhinitis is less common in the elderly, it should still be a consideration.

## Management

### Antihistamines

Antihistamines are a mainstay in treatment of allergic rhinitis. The first-generation or "older" antihistamines (e.g., chlorpheniramine, diphenhydramine) are effective in reducing sneezing, itching, and rhinorrhea. They have untoward side effects, however, that are particularly notable in the elderly patient. The elderly eliminate both first-and second-generation antihistamines more slowly [8]. The well-known side effects of the first-generation antihistamines, sedation and decreased reaction time, are more pronounced in the elderly. The anticholinergic effects are drying of the mouth and eyes, blurred vision, urinary retention, and constipation. These drugs should therefore be used cautiously in older patients and should be avoided in patients with symptomatic prostatic hypertrophy, bladder neck obstruction, and narrow angle glaucoma.

The second-generation antihistamines (loratadine, cetirizine, fexofendaine, desloratadine and levoceterizine) are much better tolerated, with little or no sedative or anticholinergic effects. Because they are metabolized more slowly in the elderly, however, one should start with a lower dose in this age group [9].

### Decongestants

Decongestants are α-adrenergic agonists that reduce nasal swelling, thus relieving congestion. The most commonly used agent is pseudoepherine. Central nervous system stimulation by these agents may result in anxiety, irritability, insomnia, and palpitations. These drugs may aggravate urinary retention in men and women with bladder neck obstruction. They should be used cautiously in the elderly and should be avoided in patients with poorly controlled hypertension, coronary artery disease, cerebral vascular disease, and bladder neck obstruction.

### Anti-inflammatory nasal sprays

These agents may be useful in reducing sneezing, itching, congestion, and rhinorrhea and are extremely safe in the elderly. They can be divided into nonsteroidal agents (cromolyn, azelastine and olopatadine) and corticosteroids (beclomethasone, flunisolide, triamcinolone, budesonide, fluticasone and ciclesonide).

### Leukotriene inhibitors

Montelukast has been approved in the United States for use in the treatment of allergic rhinitis. Although there is no study on the long-term therapeutic experience with montelukast for use in the elderly, this drug is generally safe and well tolerated. Other available leukotriene inhibitors include zafirlukast and zileuton.

### Environmental control

Reducing exposure to allergens and irritants is an important adjunct in treating patients with allergic and idiopathic rhinitis.

### Immunotherapy

If environmental measures and appropriate medications are not helping the patient with allergic rhinitis, immunotherapy (allergy injections) can be instituted; they have been shown to be highly effective.

### Non-allergic (idiopathic, vasomotor)

Idiopathic rhinitis refers to inflammation of the nasal mucous membrane unrelated to allergy, infection, structural lesions, or systemic disease. The term *vasomotor rhinitis *is frequently used, which implies that the cause in known; this is not the case, however, and therefore the preferred designation is idiopathic rhinitis [10].

The same approach to therapeutic management of allergic rhinitis applies to idiopathic rhinitis. The FDA has approved the use of intranasal azelastine for this condition.

### Drug-induced rhinitis

Over 400 brand name drugs list rhinitis as a side effect. The elderly patient is frequently being treated for a variety of medical conditions with a number of medications that can result in untoward effects of the nose. It is well known the topical decongestants that downregulate α-adrenergic receptors on nasal vasculature can cause rebound vasodilation with overuse. Older patients are at particular risk because of preexisting thinning and dryness of the nasal mucosa.

A host of antihypertensive drugs, including central adrenergic blockers (clonidine), postganglionic adrenergic blockers (guanethidine), β-adrenergic blockers (propranolol), α-adrenergic blockers (prazosin), vasodilators (hydralazine), and diuretics (hydrochlorothiazide), may cause nasal obstruction. Conjugated estrogens may also increase nasal airway resistance.

Aspirin is a well-known trigger of bronchospasm in patients with nasal polyps and asthma (Aspirin Exacerbated Respiratory Disease) but it can also cause severe rhinitis in asthmatics with and without associated polyps. The mechanism of aspirin is believed to be a cyclooxygenase block that shifts arachidonic acid metabolism to the lipoxygenase pathway, with leukotriene generation resulting in immediate nasal symptoms of rhinorrhea and obstruction.

Psychotropic drugs and Viagra, drugs likely to be used by the elderly, have also been shown to result in rhinitis.

### Nonallergic rhinitis with eosinophilia (NARES)

NARES is characterized by eosinophil infiltration of nasal tissue. Symptoms consist of perennial nasal congestion and rhinorrhea. The response of intranasal corticosteroid is generally excellent.

### Gustatory rhinitis

This condition consists of profuse rhinorrhea elicited by eating food, particularly highly seasoned foods. Cold air may also be a trigger. Anticholinergic drugs may be useful in reducing the rhinorrhea of gustatory rhinitis. Ipratropium bromide is now available in nasal spray form 0.03%, which has no systemic absorption, and as such is effective and safe.

### Atrophic rhinitis

This condition of unknown cause is often seen in the elderly. Atrophy and crusting of the nasal mucous membrane occur with resorption of the underlying bone. The crusting may result in an unpleasant odor called ozena [11]. Primary atrophic rhinitis may be caused on rare occasions by organisms such as *Klebsiella ozaenae*. Secondary atrophic rhinitis may result from nasal surgery, particularly from turbinectomy performed for nasal congestion or the previously used procedure for chronic rhinosinusititis bilateral intranasal sphenoethmoidectomy [12].

### Surgical treatment

Surgical reconstruction of the aging nose is aimed at reconstituting support for the nasal upper lateral cartilage and elevating the drooping nasal tip. Removal of turbinate mucosa should be avoided, especially when excessive dryness is already a factor [5].

Septoplasty with or without inferior turbinate reduction in patients 65 years or older with nasal septal deviation may be beneficial in this population [13].

## Appendix 1

Factors affecting medical treatment outcomes in the elderly

• Polypharmacy and compliance

• Cognitive dysfunction and memory impairment

• Alterations in body composition (muscle, fat, water)

• Impaired hepatic and renal function

• Cost and limited resources

## Appendix 2

Types of Rhinitis in the Elderly

• Allergic

• Non-allergic (idiopathic, vasomotor)

• Drug-induced

• NARES

• Gustatory

• Atrophic

## Competing interests

The author declares that he has no competing interests.
